# Benign Spontaneous Pneumomediastinum in a Late Preterm Newborn

**DOI:** 10.7759/cureus.31413

**Published:** 2022-11-12

**Authors:** Jamal M Alhudhaif, Abdullah Y Almusallam, Adel A Alfayez, Hawazin S Alqahtani, Shahad Alwazzan

**Affiliations:** 1 Pediatric Surgery, Prince Sultan Military Medical City, Riyadh, SAU; 2 Pediatric Surgery, Maternity and Children Hospital Buraydah, Buraydah, SAU; 3 General Surgery, Prince Sultan Military Medical City, Riyadh, SAU; 4 Pediatrics, King Saud Bin Abdulaziz University for Health Sciences, Riyadh, SAU

**Keywords:** pre-term, chest radiography, respiratory distress, mediastinal emphysema, pneumomediastinum

## Abstract

*Spontaneous pneumomediastinum* is defined as free air in the mediastinum without an explanation or known cause. Late preterm neonates are rarely affected; however, a known risk factor is aggressive resuscitative maneuvers. Moreover, spontaneous pneumomediastinum is a rare cause of neonatal respiratory distress diagnosed in the post-natal period using chest radiography. In contrast, preterm babies with respiratory distress syndrome are associated with pneumomediastinum. The condition is considered benign, and a conservative, non-interventional management approach is widely accepted, with complete gradual resolution as the usual course in affected neonates.

## Introduction

Pneumomediastinum or mediastinal emphysema is defined as free air in the mediastinum detected by radiography. In some cases, pneumomediastinum can be associated with life-threatening conditions such as esophageal rupture. However, in preterm babies, pneumomediastinum is a radiological finding associated with respiratory distress syndrome [1]. According to the etiology, the condition is categorized as either spontaneous, primary, or secondary. Spontaneous pneumomediastinum (SPM) is defined by the absence of an identifiable known cause. SPM is a rare, mostly benign, and self-limiting condition, commonly affecting young males and rarely newborns [2].

The incidence of SPM in a neonatal intensive care unit (NICU) is estimated to be 0.1% of all hospitalized neonates [3]. SPM can be either asymptomatic or cause significant respiratory distress [4]. As such, knowing whether SPM is causing respiratory distress or not is essential in clinical settings. We report a clinical case of a late preterm newborn, who had an uncomplicated spontaneous normal vaginal delivery, but later, following chest radiography, was found to have anterior SPM with respiratory distress, requiring invasive ventilation.

## Case presentation

**Figure 1:**
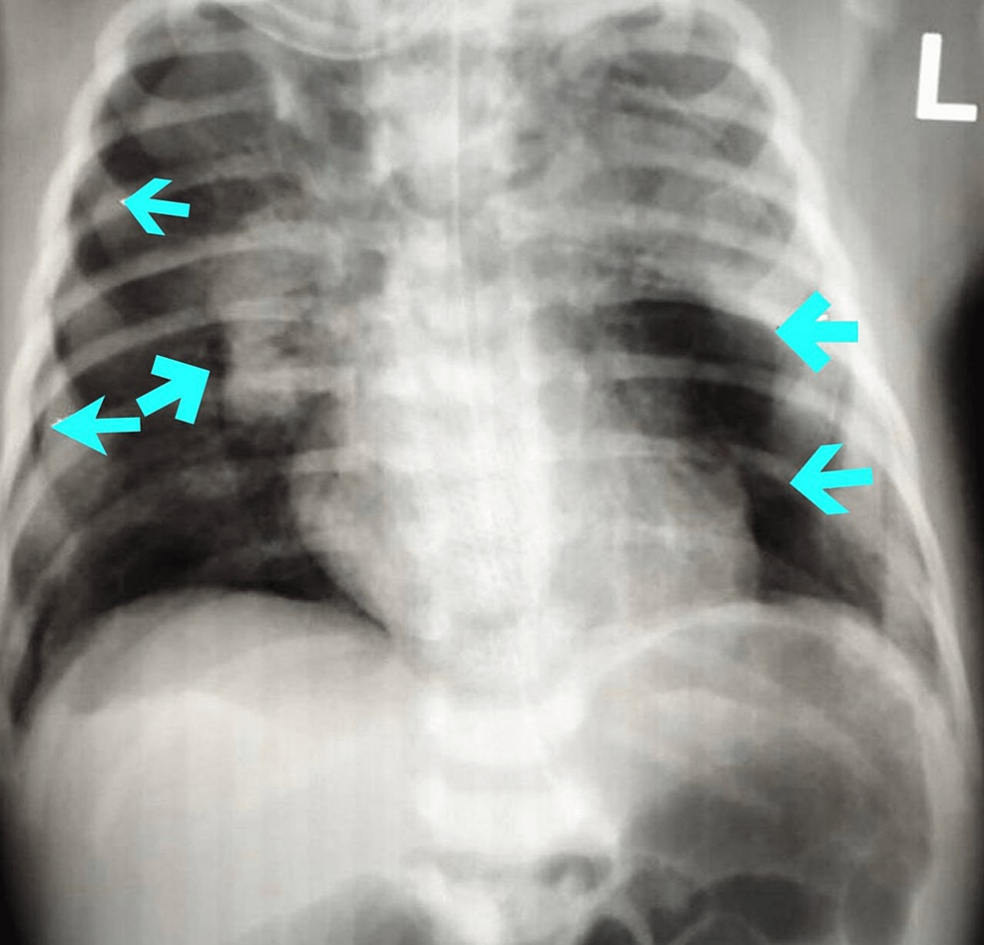
Chest X-ray, arrows demonstrating the presence of the large elliptical-shaped lucent area which is noted at the left pericardial region, causing separation and elevation of the thymic shadow, concerning pneumothorax and pneumomediastinum.

**Figure 2:**
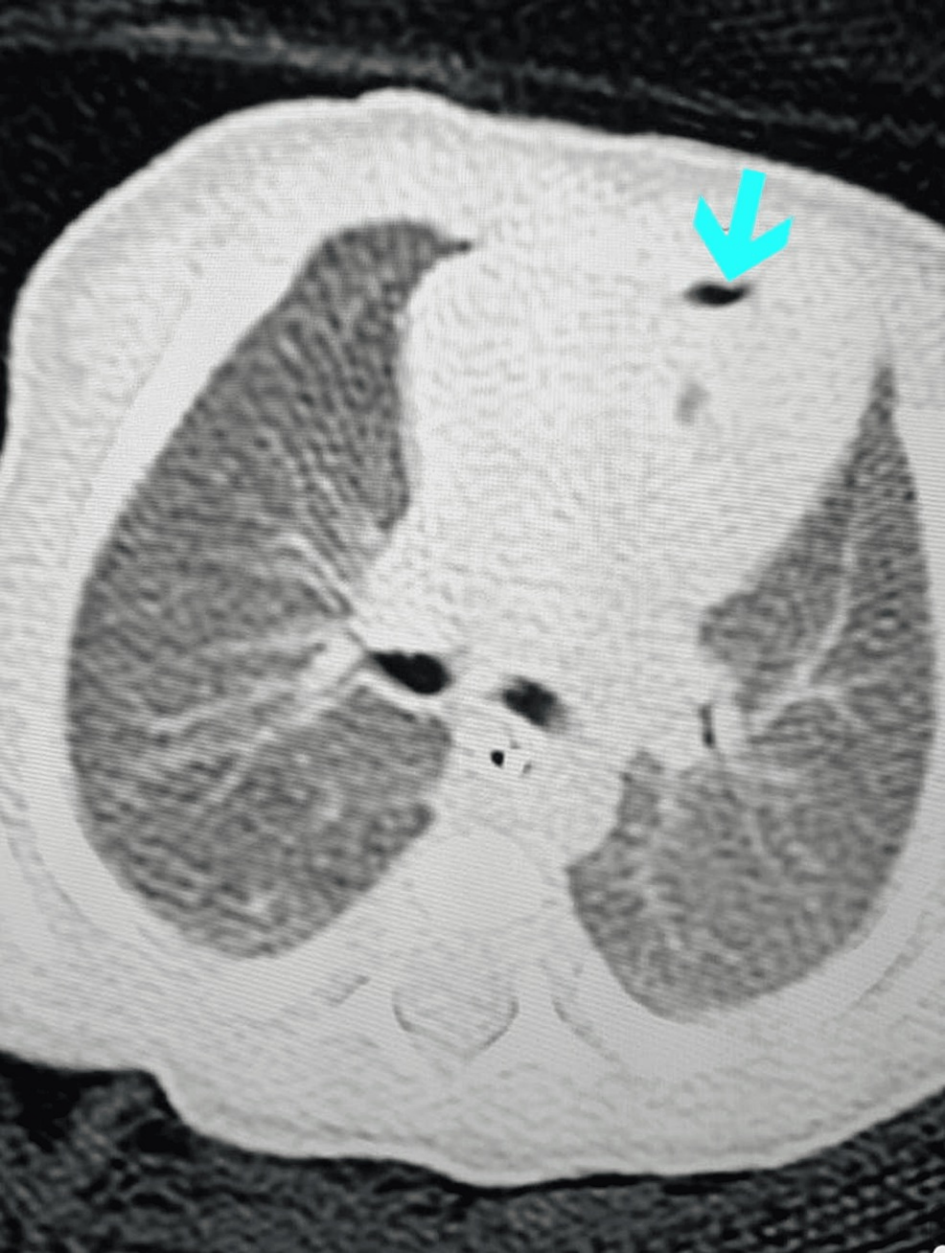
Chest (CT) scan demonstrated and revealed multiple tiny pockets of gases involving the anterior mediastinum which represents a pneumomediastinum. Grossly, there was no evidence of pericardial effusion and no pleural effusion.

A baby boy was born at 34+1 weeks gestation to a healthy 35-year-old mother via an uncomplicated spontaneous vaginal delivery at our tertiary hospital. Prenatal history is positive for preterm membrane rupture (PROM) and clear amniotic fluid with unknown group B streptococcus (GBS) status. She received a complete course of 72 hours of ampicillin before delivery. The baby's body weight at birth was 2.92 kg. Immediately after birth, the baby started to show signs of respiratory distress, and he was given 2 doses of Survanta (Beractant)*. Chest X-ray was done shortly after, which showed an endotracheal tube in a good position, and an enteric feeding tube in proper positions bilaterally increased ground-glass lung density. A large elliptical-shaped lucent area was noted at the left pericardial region, causing separation and elevation of the thymic shadow concerning pneumothorax and pneumomediastinum (figure 1). Hence, pediatric surgery services were consulted regarding this for further management. After that, he was shifted to the Neonatal Intensive Care Unit (NICU) for further management, observation of his Respiratory Distress Syndrome (RDS), and to rule-out sepsis. In (NICU) he was on Continuous Positive Airway Pressure (CPAP), then intubated with synchronized intermittent mandatory ventilation with a rate of 30, a positive end-expiratory pressure (PEEP) of 25/5, and a fraction of inspired oxygen (FIO2) of 92%. Group B streptococcus cultures were established, and ampicillin and gentamycin antibiotics were started. Moreover, a chest CT scan revealed a patent trachea, major bronchi, and multiple tiny pockets of gases seen mainly within the anterior mediastinum representing a pneumomediastinum (figure 2). Grossly, there was no significant lymphadenopathy, no evidence of pericardial effusion, and no pleural effusion. We choose the non-interventional approach as the condition is self-limiting, which warrants conservative management. The baby was extubated on the second day, and whether the respiratory distress syndrome vs. the presence of free air in the mediastinum would explain his respiratory distress status is unknown. After 10 days of closely monitoring the baby in NICU, he was seen and examined, active, tolerating his full feeding of 49 ml every 3 hours, and with adequate urine output of 3 ml/kg/day. On examination, he was hemodynamically stable, and the temperature was 36.9^o^c, HR:144 RR:52, O2S:97-100 saturating well on room air, with good primitive reflexes. Auscultation of chest and heart sounds was unremarkable, and abdominal examination was not suggestive of any concerns or organomegaly. A few days after he was extubated, Repeated chest radiography showed complete resolution of the pneumomediastinum. The patient was discharged in good health with a two-week follow-up appointment in the outpatient clinic. Upon follow-up, he was doing fine, thriving well, tolerating breastfeeding, and with no active or new complaints.

## Discussion

Laennec first described pneumomediastinum in 1819 as a traumatic cause explaining the presence of free air in the mediastinum [5]. Hamman later described SPM in 1939. Respiratory distress syndrome (hyaline membrane disease) is a common condition in preterm babies that explains the occurrence of pneumomediastinum. The pathophysiology was explained by Macklin, such that the alveoli ruptured due to overinflation or pressure gradient differences between the alveoli and the interstitial space, allowing air to dissipate into the mediastinum [1]. According to Steele et al. in 1971, pneumomediastinum is not considered a common cause of neonatal respiratory distress, with an incidence of approximately 2.5 per 1000 live births and a percentage of 2.3% of normal deliveries [6]. In neonates, SPM is occasionally associated with risk factors such as prematurity, surfactant deficiency management, meconium aspiration, and pneumonia. However, it can also be seen in patients with no predisposing factors [7]. The reported cases in the literature showed that SPM is more familiar with caesarian section deliveries, while it is uncommon with uncomplicated vaginal deliveries. This is explained by the changes in physiological pressure or delivery process until post-natal life immediately after delivery [8,9]. In our case, the patient was a product of an uncomplicated spontaneous vaginal delivery, in which radiography demonstrated pneumomediastinum immediately after birth. Patients with SPM may be asymptomatic; however, in some cases, symptoms such as respiratory distress, grunting, and chest pain have been reported [1,4]. Our patient presented with post-natal respiratory distress first diagnosed and managed as respiratory distress syndrome, along with free air in the anterior mediastinum.

As published in some reports, the diagnosis of SPM can be clinically diagnosed by physiological changes in respiratory setting requirements, respiratory distress, and subcutaneous emphysema [1]. Other publications illustrated that auscultation of the patient's chest could reveal Hamman's sign (a crunching sound with systole), considered a pathognomonic finding of this condition [10]. Chest radiography is the most common diagnostic modality used in the literature. The radiographic signs seen particularly in anterior pneumomediastinum were first described by Moseley, who stated that "on the frontal projection, air may be seen to extend to both sides or localize on one side, elevating the thymic lobes and giving rise to a crescentic configuration akin to a windblown spinnaker sail," and the continuous diaphragmatic sign [11]. Chest CT is useful in doubtful, unclear chest X-ray findings and helps delineate further radiological findings by ruling out the presence of other pathologies [1]. The prognosis of patients with SPM is excellent, and the condition resolves gradually and completely with conservative management, including rest, minimal resuscitative maneuvers (if clinically permitted), and analgesia as needed.

## Conclusions

Spontaneous pneumomediastinum in preterm neonates is mainly associated with respiratory distress syndrome. Aggressive resuscitation maneuvers would contribute to this. The etiology behind it is the alveolar rupture due to overinflation or weak alveolar sacs, further allowing air to leak outside the sacs. Detailed history taking, physical examination, and chest x-ray are mainly diagnostic. Ultrasound could be used in NICU for evaluation of abnormal mediastinal radiolucency. The condition carries a good prognosis and is known to be self-limiting, which would be resolved without any intervention.
